# Burden and severity of children's hospitalizations by respiratory syncytial virus in Portugal, 2015–2018

**DOI:** 10.1111/irv.13066

**Published:** 2022-11-14

**Authors:** Teresa Bandeira, Mafalda Carmo, Hugo Lopes, Catarina Gomes, Margarida Martins, Carlos Guzman, Mathieu Bangert, Fernanda Rodrigues, Gustavo Januário, Teresa Tomé, Inês Azevedo

**Affiliations:** ^1^ Centro Hospitalar Universitário Lisboa Norte, Faculdade de Medicina Universidade de Lisboa, CAML Lisbon Portugal; ^2^ IQVIA Barcelona Spain; ^3^ IQVIA Lisbon Portugal; ^4^ Sanofi Lisbon Portugal; ^5^ Sanofi Madrid Spain; ^6^ Sanofi Lyon France; ^7^ Hospital Pediátrico Centro Hospitalar e Universitário de Coimbra Coimbra Portugal; ^8^ Faculdade de Medicina Universidade de Coimbra Coimbra Portugal; ^9^ Centro Hospitalar Universitário Lisboa Central Lisbon Portugal; ^10^ Faculdade de Medicina Universidade do Porto, Centro Hospitalar Universitário S. João; EPIUnit, Instituto de Saúde Pública Porto Portugal

**Keywords:** administrative data, burden, hospitalizations, lower respiratory infection, Portugal, respiratory syncytial virus

## Abstract

**Background:**

Respiratory syncytial virus (RSV) is a leading cause of acute lower respiratory infection (ALRI) in young children and is of considerable burden on healthcare systems. Our study aimed to evaluate ALRI hospitalizations related to RSV in children in Portugal.

**Methods:**

We reviewed hospitalizations potentially related to RSV in children aged <5 years from 2015 to 2018, using anonymized administrative data covering all public hospital discharges in mainland Portugal. Three case definitions were considered: (a) RSV‐specific, (b) (a) plus unspecified acute bronchiolitis (RSV‐specific & Bronchiolitis), and (c) (b) plus unspecified ALRI (RSV‐specific & ALRI).

**Results:**

A total of 9697 RSV‐specific hospitalizations were identified from 2015 to 2018—increasing to 26 062 for RSV‐specific & ALRI hospitalizations—of which 74.7% were during seasons 2015/2016–2017/2018 (November–March). Mean hospitalization rates per season were, for RSV‐specific, RSV‐specific & Bronchiolitis, and RSV‐specific & ALRI, respectively, 5.6, 9.4, and 11.8 per 1000 children aged <5 years and 13.4, 22.5, and 25.9 in children aged <2 years. Most RSV‐specific hospitalizations occurred in healthy children (94.9%) and in children aged <2 years (96.3%). Annual direct costs of €2.4 million were estimated for RSV‐specific hospitalizations—rising to €5.1 million for RSV‐specific & ALRI—mostly driven by healthy children (87.6%).

**Conclusion:**

RSV is accountable for a substantial number of hospitalizations in children, especially during their first year of life. Hospitalizations are mainly driven by healthy children. The variability of the potential RSV burden across case definitions highlights the need for a universal RSV surveillance system to guide prevention strategies.

## 1 INTRODUCTION

Respiratory syncytial virus (RSV) is responsible for seasonal diseases causing a great burden on healthcare systems across the world.^1^ RSV is the leading cause of acute lower respiratory infection (ALRI) in children and a major driver of childhood hospitalizations.^2^, ^3^, ^4^, ^5^ The clinical manifestations can range from mild to severe respiratory infections, including bronchiolitis and pneumonia.^1^ Globally, 33.0 million RSV‐associated ALRI episodes were estimated to have occurred in children <5 years in 2019, causing around 3.6 million RSV‐associated ALRI hospital admissions, 26,300 RSV‐associated ALRI in‐hospital deaths, and 101,400 RSV‐attributable overall deaths.^5^ Nearly 20% of the infections, 39% of the hospitalizations, 51% of the in‐hospital mortality, and 45% of the total RSV‐attributable overall deaths across the world occurred in infants younger than 6 months.^5^ Preterm infants or children with underlying medical conditions are more likely to suffer from serious disease and have higher rates of morbidity and mortality.^1^ Furthermore, children with severe RSV infection early in life are at greater risk of developing subsequent wheezing, hyperreactive airway disease, and asthma in later childhood.^1^, ^6^, ^7^

Current prevention strategies are focused on specific at‐risk children, despite evidence that most hospitalizations for RSV occur in previously healthy children born at term.^8^, ^9^, ^10^, ^11^, ^12^ The development of RSV vaccines and immunoprophylactic agents^13^ has given a new impetus to research on the pediatric RSV burden across the world, particularly on severe (hospitalized) cases, as more evidence is required to prepare their introduction to health systems. Particularly, a more granular understanding of the true burden in the populations that may benefit the most from preventive measures is required to prevent these frequently severe hospitalizations and short‐term healthcare episodic burdens, as well as associated long‐term respiratory morbidity.^7^, ^13^

In Portugal, there are no recent studies on the burden of RSV‐related hospitalizations, using the International Classification of Diseases, Tenth Revision, Clinical Modification (ICD‐10 CM), which has introduced more RSV‐specific diagnosis codes. Furthermore, there are no studies assessing RSV‐related hospitalizations in children between 2 and 5 years. The most comprehensive study covers acute bronchiolitis in children aged <2 years.^14^, ^15^ Recent evidence suggests that combining RSV with other unspecified ALRI diagnoses may help overcome the obstacle of underestimating RSV cases in children aged <5 years, without sacrificing high specificity, particularly in hospitals with lower virus identification resources—as cases are often coded according to clinical manifestations but without specifying the infectious agent causing the disease.^3^, ^16^, ^17^ Indeed, Cai et al.^16^ showed that RSV‐specific ICD‐10 codes alone had high specificity (99.8%) and poor sensitivity (6%) but, when combined with general ALRI ICD‐10 codes, the specificity remained high (>90%) and the sensitivity increased (44%). Hamilton et al. have also identified this combination of ICD‐10 codes as the top‐performing algorithm to identify RSV cases.^18^

In our study, we aimed to describe the burden of potentially RSV‐related hospitalizations in Portuguese mainland public hospitals, in children under 5 years of age, from epidemic seasons 2015 to 2018, and analyze the incidence and costs of the hospitalizations for the National Health System (NHS). Secondary objectives included the analysis of RSV seasonality, the description of the clinical and demographic characteristics of hospitalized patients, and the outcomes and severity indicators of the episodes.

## 2 METHODS

### 2.1 Study design

Anonymized administrative data on hospitalizations potentially due to RSV in children aged <5 years (January 2015–December 2018) were reviewed. The data were provided by the *Administração Central do Sistema de Saúde* (ACSS), which collects administrative and clinical data for all hospitalization episodes in Portuguese public hospitals, including information on diagnoses and procedures performed during hospital stay, which are coded using the ICD‐9 CM and ICD‐10 CM/PCS.

### 2.2 RSV case definition

A child was classified as having an RSV‐related event based on hospital discharge ICD‐9/10 codes, if coded as a primary or secondary diagnosis, according to three distinct case definitions (Table 1): (a) RSV‐specific, (b) RSV‐specific and unspecified acute bronchiolitis (RSV‐specific & Bronchiolitis), and (c) RSV‐specific and unspecified ALRI (RSV‐specific & ALRI). Unless otherwise stated, results are presented for the RSV‐specific definition. Case definitions were based on findings from Cai et al.^16^

**TABLE 1:** List of ICD‐9/10 codes used in each case definition and frequency of observation of each code per year, in children aged <5 years, in any primary or secondary diagnosis field, in Portuguese public hospitals, between 1st January 2015–31st December 2018

			Description	ICD‐9	ICD‐10	2015^a^	2016^a^	2017^a^	2018^a^	Total 2015–18^a^
**RSV‐specific & ALRI**	**RSV‐specific & Bronchiolitis**	**RSV‐specific**	**RSV**	079.6	NA	136	181	1	‐	**318**
			**Acute bronchiolitis due to RSV**	466.11	J21.0	1945	2252	2384	1988	**8569**
			**Pneumonia due to RSV**	480.1	J12.1	126	137	159	109	**531**
			**RSV as the cause of diseases classified elsewhere**	NA	B97.4	‐	16	114	110	**240**
			**Acute bronchitis due to RSV**	NA	J20.5	‐	8	23	58	**89**
			**Acute bronchiolitis due to other infectious organisms**	466.19	J21.8	1945	2268	2498	2098	**8809**
			**Acute bronchiolitis, unspecified**		J21.9					
			**Acute bronchitis due to other infectious organisms**	466.0	J20.8	206	204	144	172	**726**
			**Acute bronchitis, unspecified**		J20.9					
			**Unspecified ALRI**	519.8	J22	504	420	425	428	**1777**
			**Viral pneumonia, not elsewhere classified**	480.3;480.8; 480.9; 487.0	J12.8; J12.9	217	199	111	126	**653**
			**Pneumonia, organism unspecified**	485; 486	J18.0, J18.8, J18.9	1405	1239	1092	1082	**4818**

Abbreviations: ALRI, acute lower respiratory infection; N/A, not applicable; RSV, respiratory syncytial virus.

^a^ The table contains the number of hospitalization episodes codified with each code, as either a primary or secondary diagnosis. The total frequency is higher than the number of episodes, as one episode may have had several diagnosis codes associated.

Total hospitalizations excluded admissions related to routine birth (ICD‐9 CM codes V30.x through V39.x or ICD‐10 CM Z38 as a primary diagnosis). All episodes with a length of hospital stay (LOS) of less than 24 h are not included in the database.

The unit of analysis (case) was the hospitalization episode. This means that a patient who was admitted to the hospital multiple times was accounted for each time separately. An exception was made for the analysis of mean cost per patient, where the cost of all included cases was divided by the number of unique patients.

### 2.3 RSV season

To increase accuracy for RSV potential cases, as is described in temperate climates,^2^, ^19^ data were analyzed by epidemic season (November to March^20^) in 2015/2016, 2016/2017, and 2017/2018, except for the analyses of date of birth and weekly evolution of RSV cases, which were done for the total study population.

### 2.4 Risk factors

The additional ICD‐9/10 codes for diagnostic information were used to identify children who had at least a risk factor for severe RSV (Table S1), in any primary or secondary diagnosis field. The following risk factors were considered to classify children as having a risk factor for RSV: heart disease, neuromuscular disorders, bronchopulmonary dysplasia, Down syndrome, immunodeficiency, congenital disorders of the respiratory system, congenital musculoskeletal anomalies, and cystic fibrosis. Prematurity, low birth weight, and exposure to tobacco were separately assessed as they do not correspond to underlying medical conditions, and thus, the likelihood of not being inserted by medical coders, particularly after the first hospitalization, is high.^14^

### 2.5 RSV‐related mortality

In‐hospital mortality was evaluated based on discharge status and corresponds to the hospitalization episodes classified as RSV related, which resulted in in‐hospital death during the respective episode. No definitive causal link to RSV infection can be ascertained from this study.

### 2.6 Other study definitions

Hospital admission rates are presented as cases per 1000 population, for both genders combined, expressed as an annual rate. The population denominator for calculation of the years in analysis was obtained from INE—*Instituto Nacional de Estatística* (National Statistics Institute). For the population aged <1 year, the denominator values were estimated by the mean number of monthly live births from the corresponding year multiplied by the proportion of months in the respective age group (e.g., multiplied by 0.1 for children aged <1 month).^14^ LOS was calculated from admission to the hospital to discharge.

Mechanical ventilation was used as a surrogate indicator of intensive care unit (ICU) admissions, as these are not separately identified in the database.^14^ Additionally, a respiratory severity marker was created, combining the following procedures and diagnosis: supplementary oxygen therapy, hypoxemia, invasive and noninvasive ventilation, respiratory failure, and other abnormalities of breathing (codes detailed in Table S2). Oxygen therapy is recommended in Portugal when blood oxygen saturation and pulse (SpO_2_) is ≤92%,^21^ a level that may be considered a predictor of clinical deterioration.^22^

### 2.7 Costs

Only direct costs were estimated, using a diagnosis‐related group (DRG)‐based budget allocation model. Hospitalization costs were computed by multiplying each cost weight, considering the DRG of each episode, with the Portuguese fixed cost multiplier and funding price applicable for the 2018 year, as defined by the ACSS.

### 2.8 Statistical analysis

Cases were stratified by age and the presence of at least one risk factor for RSV infection. The following age subgroups were considered: 0–1, 1–2, 2–3, 3–6, 6–12, 12–24, 24–36, and 36–60 months. Continuous data are presented as mean (standard deviation, SD) and/or median (interquartile range, IQR), as appropriate. The statistical analyses were carried out using IBM SPSS Statistics 19.0.

### 2.9 Ethical considerations

Data were provided anonymized from ACCS and may be used for research purposes without ethics committee approval or informed consent.

## 3 RESULTS

### 3.1 Seasonality

**FIGURE 1:**
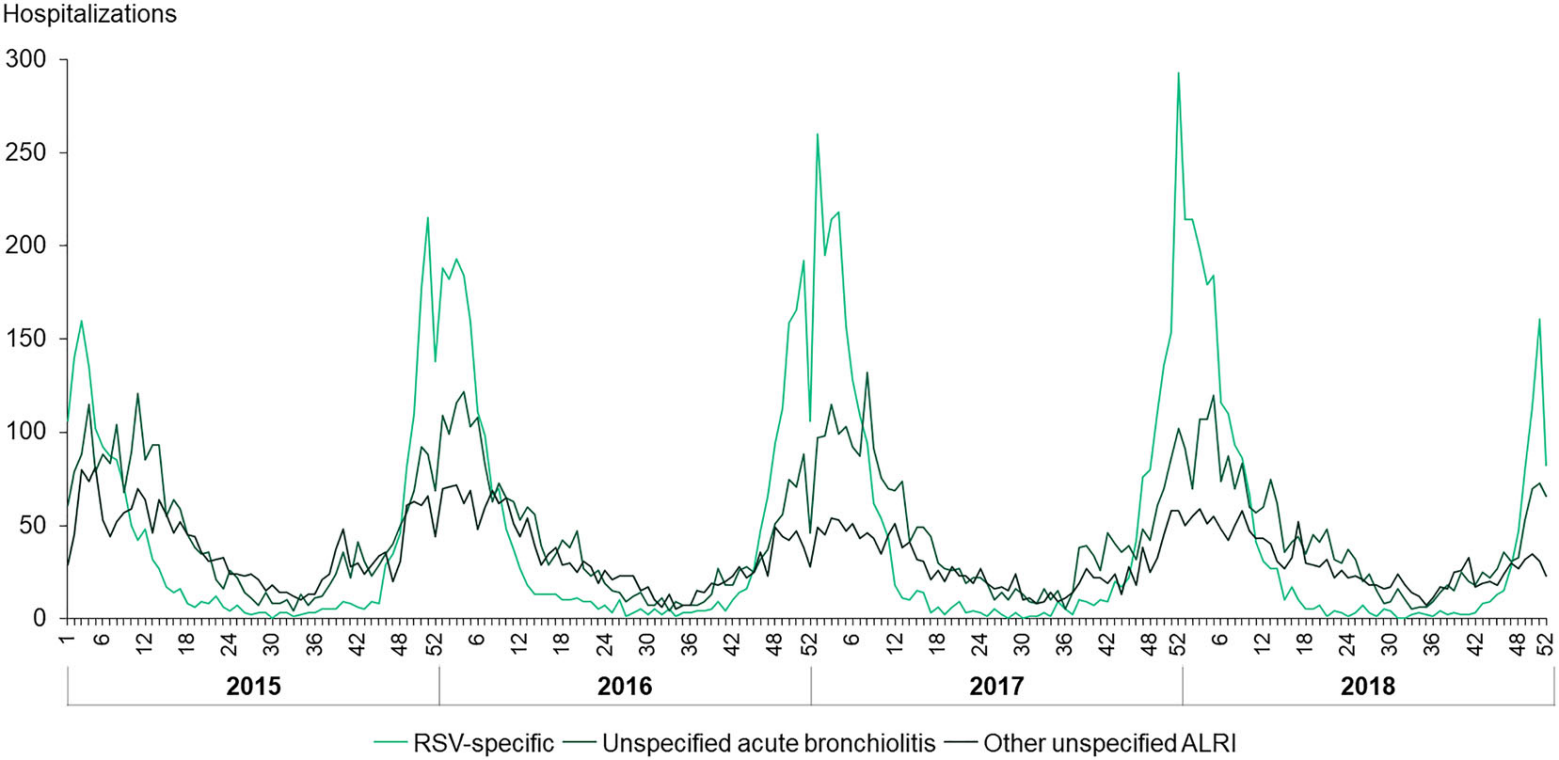
Weekly respiratory syncytial virus‐related hospitalizations in children <5 years of age, per group of ICD‐9/10 codes, in Portuguese public hospitals, between January 1, 2015, and December 31, 2018. ALRI, acute lower respiratory infection; RSV, respiratory syncytial virus

A total of 9697 RSV‐specific, 19 118 RSV‐specific & Bronchiolitis, and 26 062 RSV‐specific & ALRI hospitalizations were identified between 2015 and 2018, of which 74.7%, 63.4%, and 58.4% occurred during epidemic seasons 2015/2016 to 2017/2018 (November to March), respectively. These 26 062 RSV‐specific & ALRI cases represented 6.7% of all‐cause hospitalizations in children aged <5 years and 23.4% after exclusion of hospitalizations related to routine birth. The maximum number of cases was registered in week 52 of season 2017/2018 across all definitions (Figure 1).

**FIGURE 2:**
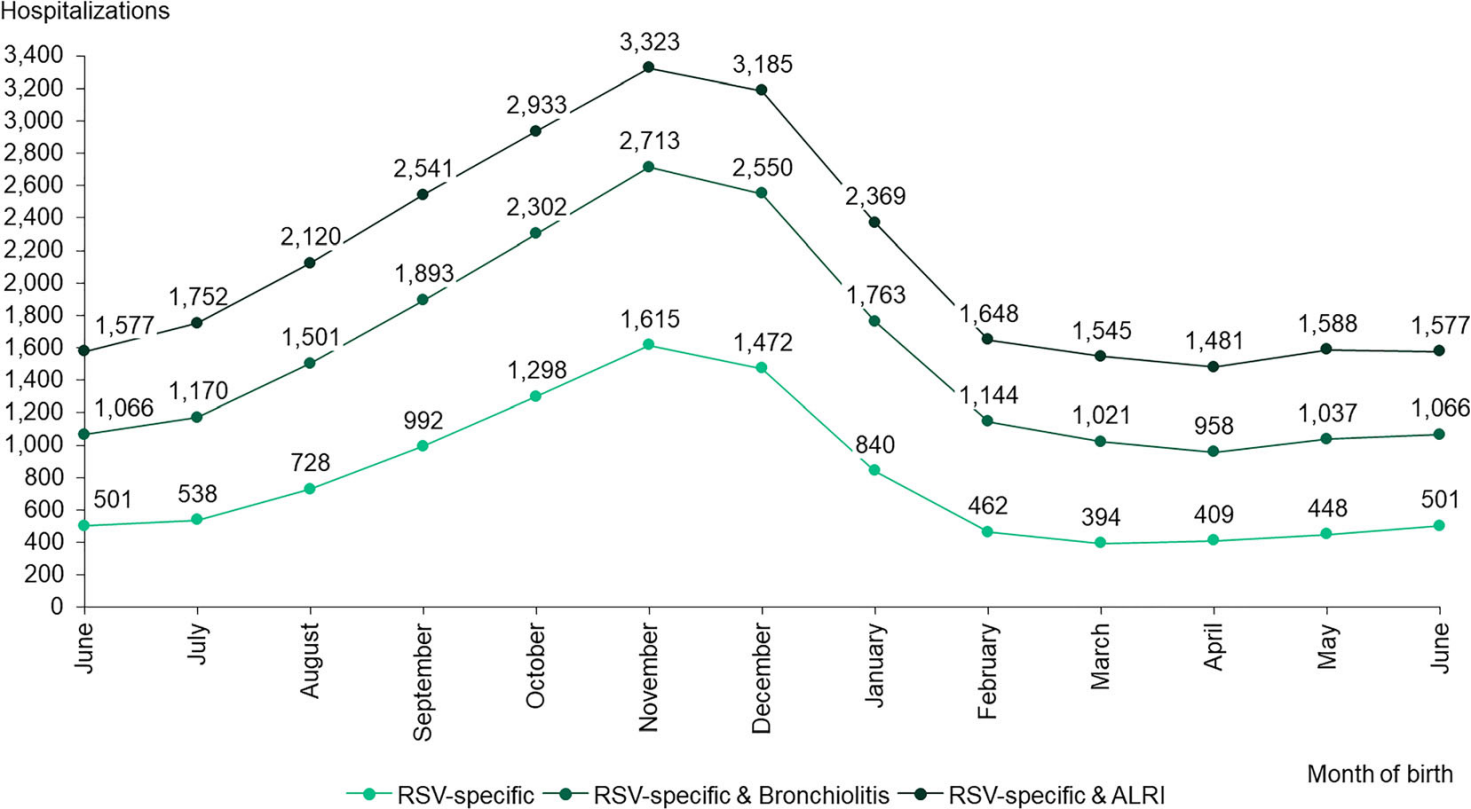
Respiratory syncytial virus‐related hospitalizations in children aged <5 years, per month of birth, in Portuguese public hospitals, between January 1, 2015, and December 31, 2018. ALRI, acute lower respiratory infection; RSV, respiratory syncytial virus

Considering the date of birth of hospitalized children, the peak number of cases was observed in children who were born in November across all definitions (Figure 2).

**FIGURE 3:**
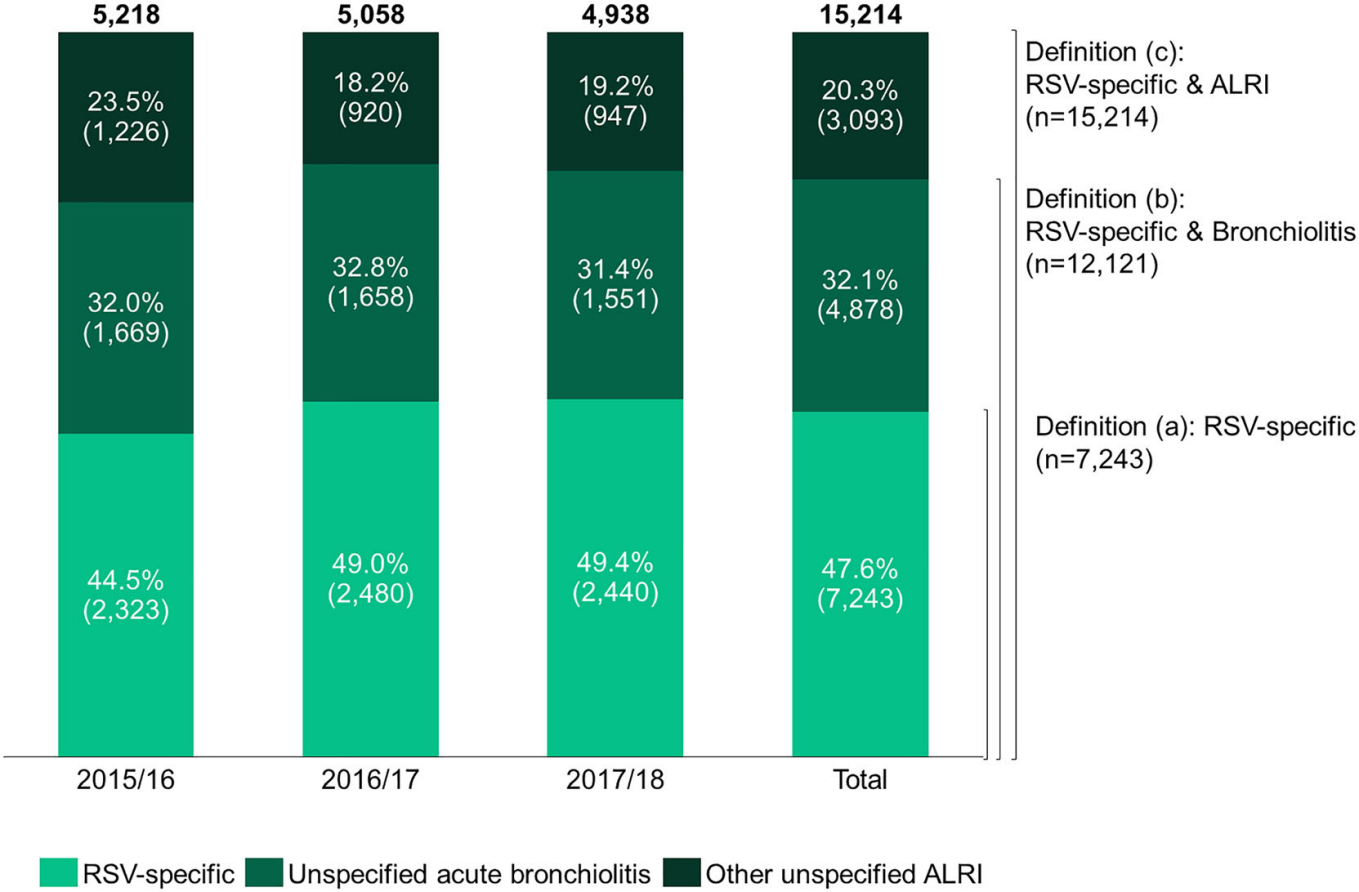
Respiratory syncytial virus‐related hospitalizations in children aged <5 years, per groups of ICD‐9/10 codes, per season, in Portuguese public hospitals. ALRI, acute lower respiratory infection; RSV, respiratory syncytial virus

Figure 3 details the number of cases in each of the analyzed seasons for each definition. A total of 15 214 RSV‐specific & ALRI cases were observed during the three analyzed epidemic seasons, of which 5218 (34.3%), 5058 (33.2%), and 4938 (32.5%) in 2015/2016, 2016/2017, and 2017/2018, respectively. RSV‐specific codes accounted for 47.6% of these cases, unspecified acute bronchiolitis codes for 32.1%, and other unspecified ALRI codes for 20.3% (Figure 3). As case definitions are cumulative, 7243 cases were classified as RSV‐specific (definition a), 12,121 as RSV‐specific & Bronchiolitis (definition b), and 15,214 as RSV‐specific & ALRI (definition c).

### 3.2 Hospitalization rate

Annual RSV‐specific hospitalization rate during the analyzed epidemic seasons was 23.8 per 1000 children <12 months of age, 3.0 per 1000 children between 12 and 23 months of age, and 0.4 per 1000 children between 24 and 49 months of age. The highest rates were observed in children aged <3 months of age, namely, 50.6 per 1000 children aged below 1 month of age, 59.4 per 1000 children between 1 and 2 months of age, and 46.2 per 1000 children between 2 and 3 months of age (Table 2).

**TABLE 2:** Estimated respiratory syncytial virus‐associated hospitalizations in patients aged <5 years, in absolute and per 1000 children, by age group, presence of at least one risk factor, and epidemic season in Portuguese public hospitals between 2015/2016 and 2017/2018

Months of age/presence of risk factors	RSV‐specific			RSV‐specific & Bronchiolitis			RSV‐specific & ALRI		
	Cases^a^	%	IR^b^	Cases^a^	%	IR^b^	Cases^a^	%	IR^b^
0–1	1101	15.2	50.6	1516	12.5	69.6	1583	10.4	72.7
**1–2**	1294	17.9	59.4	1985	16.4	91.2	2072	13.6	95.1
**2–3**	1007	13.9	46.2	1561	12.9	71.7	1628	10.7	74.8
**3–6**	1460	20.2	22.3	2461	20.3	37.7	2669	17.5	40.9
**6–12**	1321	18.2	10.1	2579	21.3	19.7	3068	20.2	23.5
**12–24**	790	10.9	3.0	1577	13.0	6.1	2430	16.0	9.4
**24–36**	167	2.3	0.7	295	2.4	1.2	879	5.8	3.4
**36–60**	103	1.4	0.2	147	1.2	0.3	885	5.8	1.7
**0–24**	6973	96.3	13.4	11 679	96.4	22.5	13 450	88.4	25.9
**24–60**	270	3.7	0.4	442	3.6	0.6	1764	11.6	2.3
**With risk factor** ^c^	366	5.1	N/A	610	5.0	N/A	912	6.0	N/A
**Without risk factor** ^c^	6877	94.9	N/A	11 511	95.0	N/A	14 302	94.0	N/A
**Total**	7243	100	5.6	12 121	100.0	9.4	15 214	100.0	11.8

Abbreviations: ALRI, acute lower respiratory infection; IR, incidence rate; N/A, not available (due to lack of data for the denominator); RSV, respiratory syncytial virus.

^a^ Total cases identified through the studied RSV seasons, namely, from November 2015 to March 2016, from November 2016 to March 2017, and from November 2017 to March 2018.

^b^ Mean annual incidence rate of RSV cases per 1000 children, considering the mean annual cases identified through the studied RSV seasons (namely, from November 2015 to March 2016, from November 2016 to March 2017, and from November 2017 to March 2018) and the mean yearly population in the respective age group.

^c^ The following risk factors were considered to classify children as having a risk factor for RSV: heart disease, neuromuscular disorders, bronchopulmonary dysplasia, Down syndrome, immunodeficiency, congenital disorders of the respiratory system, congenital musculoskeletal anomalies, and cystic fibrosis. Prematurity, low birth weight, and exposure to tobacco were not included.

### 3.3 Demographic characteristics

Of the 7243 RSV‐specific hospitalizations in epidemic seasons, 3903 (53.9%) occurred in males. The male‐to‐female ratio was 1.2. Children aged <2 years accounted for 96.3% of cases, and 67.1% of all hospitalizations occurred during the first 6 months of age. The median age for RSV‐specific hospitalization was 3.2 months (1.6–7.6 months). The results for all RSV case definitions can be found in Table 2.

### 3.4 Presence of underlying medical conditions considered as risk factors

Overall, in epidemic seasons, 6877 (94.9%) of the RSV‐specific cases were observed in children without known predisposing risk factors for severe RSV infection. Underlying medical conditions were present in 366 (5.1%) of the RSV‐specific cases (Table 2), of which 322 (4.6%) cases were in children aged <24 months and 44 (16.3%) in children aged between 24 and 59 months. Heart disease was the most common underlying disease (3.0%) in RSV‐specific cases, followed by neuromuscular disorders (1.2%), congenital disorders of the respiratory system (0.3%), Down syndrome (0.3%), bronchopulmonary dysplasia (0.3%), congenital musculoskeletal anomalies (0.3%), immunodeficiency (0.1%), and cystic fibrosis (0.0%) (Figure S1).

### 3.5 Relevant medical history associated with the child's birth

Prematurity was registered as a diagnosis in 1.2% of the RSV‐specific cases (reaching a maximum of 3.4% in the age group of 0–1 month), low birth weight in 0.7% of the cases, and exposure to tobacco in 1.5% of the cases.

### 3.6 Length of stay

Mean (SD) and median (IQR) LOS per episode are presented in Table 3. The median (IQR) LOS for RSV‐specific hospitalization was 5.0 (3.0–7.0) days. Across all definitions, the highest LOS was observed in children with a risk factor and in children under 1 month of age.

**TABLE 3:** Descriptive statistics on length of stay and costs per patient of respiratory syncytial virus‐related hospitalizations in patients aged <5 years, by age group and presence or absence of at least one risk factor, in Portuguese public hospitals between 2015/2016 and 2017/2018

Months of age/presence of risk factors	RSV‐specific			RSV‐specific & Bronchiolitis					RSV‐specific & ALRI			
	LOS (days)		Cost/patient (€)^a^	LOS (days)			Cost/patient (€)^a^		LOS (days)		Cost/patient (€)^a^	
	Mean (SD)	Median (IQR)	Mean (SD)	Median (IQR)	Mean (SD)	Median (IQR)	Mean (SD)	Median (IQR)	Mean (SD)	Median (IQR)	Mean (SD)	Median (IQR)
**0–1**	7.8 (8.7)	6 (4–9)	1447 (2873)	652 (652–948)	7.5 (8.6)	6 (4–9)	1351 (2636)	652 (652–948)	8.3 (14.4)	6 (4–9)	1664 (4820)	652 (652–948)
**1–2**	5.8 (4.2)	5 (3–7)	1054 (1757)	652 (652–652)	5.5 (4)	5 (3–7)	1008 (1622)	652 (652–652)	5.6 (5.4)	5 (3–7)	1066 (1994)	652 (652–652)
**2–3**	5.7 (3.7)	5 (3–7)	1013 (2207)	652 (652–652)	5.4 (3.9)	5 (3–7)	947 (1880)	652 (652–652)	5.5 (4.5)	5 (3–7)	969 (1872)	652 (652–652)
**3–6**	5.4 (3.7)	5 (3–7)	850 (1190)	652 (652–652)	5.1 (3.7)	4 (3–6)	837 (1062)	652 (652–652)	5.3 (5.1)	4 (3–6)	886 (1308)	652 (652–652)
**6–12**	5.3 (3.9)	5 (3–6)	836 (1583)	652 (652–652)	4.9 (3.8)	4 (3–6)	827 (1319)	652 (652–652)	5.1 (4.7)	4 (3–6)	910 (1955)	652 (652–880)
**12–24**	5.3 (4.4)	4 (3–6)	845 (1035)	652 (652–652)	4.6 (3.6)	4 (3–6)	791 (812)	652 (652–652)	4.8 (3.9)	4 (3–6)	919 (1092)	652 (652–925)
**24–36**	6.3 (7.5)	4 (3–7)	1081 (2154)	652 (652–880)	5.1 (6)	4 (2–6)	939 (1645)	652 (652–813)	4.8 (5.1)	4 (3–6)	1072 (1566)	925 (703–925)
**36–60**	6.6 (8.4)	4 (3–7)	1056 (1522)	652 (652–891)	5.7 (7.3)	4 (3–6)	942 (1281)	652 (652–880)	5 (5)	4 (3–6)	1143 (1315)	925 (880–925)
**0–24**	5.9 (5.1)	5 (3–7)	1001 (1872)	652 (652–652)	5.4 (4.8)	4 (3–7)	939 (1600)	652 (652–652)	5.6 (6.7)	4 (3–7)	1027 (2292)	652 (652–880)
**24–60**	6.4 (7.9)	4 (3–7)	1071 (1941)	652 (652–880)	5.3 (6.5)	4 (2–6)	940 (1534)	652 (652–874)	4.9 (5)	4 (3–6)	1107 (1447)	925 (880–925)
**With risk factor** ^b^	11.4 (14.6)	7 (4–12)	2504 (5343)	880 (652–1583)	10 (12.5)	7 (4–11)	2003 (4319)	852 (652–1323)	11.2 (19.8)	6 (4–11)	2692 (7214)	925 (652–1688)
**Without risk factor** ^b^	5.6 (3.9)	5 (3–7)	926 (1443)	652 (652–652)	5.2 (3.9)	4 (3–6)	883 (1285)	652 (652–652)	5.1 (4.3)	4 (3–6)	933 (1331)	652 (652–880)
**Total**	5.9 (5.2)	5 (3–7)	1004 (1875)	652 (652–652)	5.4 (4.9)	4 (3–7)	939 (1598)	652 (652–652)	5.5 (6.6)	4 (3–7)	1036 (2211)	652 (652–925)

Abbreviations: ALRI, acute lower respiratory infection; IQR, interquartile range; LOS, length of stay; RSV, respiratory syncytial virus; SD, standard deviation.

^a^ Refers to the costs of all hospitalizations associated with the same patient. In case the same patient had more than one hospitalization with different ages, comorbidities status, or RSV classifications, these were analyzed as two separate patients for the purpose of this analysis.

^b^ The following risk factors were considered to classify children as having a risk factor for RSV: heart disease, neuromuscular disorders, bronchopulmonary dysplasia, Down syndrome, immunodeficiency, congenital disorders of the respiratory system, congenital musculoskeletal anomalies, and cystic fibrosis. Prematurity, low birth weight, and exposure to tobacco were not included.

### 3.7 Respiratory severity marker

A respiratory severity marker was reported in 5193 (71.7%) of the RSV‐specific cases. Invasive mechanical ventilation was used in 106 (1.5%) cases, noninvasive ventilation in 591 (8.2%), and oxygen supplementation in 4209 (58.1%). Results for all RSV case definitions can be found in Figure S2.

### 3.8 Mortality

During the study period, nine deaths among the 7243 RSV‐specific cases resulted in an in‐hospital mortality rate of 0.1%, with eight out of the nine deaths occurring in children aged <2 years. In children with a risk factor, a mortality rate of 1.4% was observed. The mortality rate in the RSV‐specific & ALRI cases was 0.2%, with 22 out of 29 deaths occurring in children aged <2 years. The median age of children who died during an RSV‐specific hospitalization was 4.4 months (0.2–11.0). Children who died during an RSV‐specific & ALRI hospitalization presented a mean age of 11.0 months (0.5–16.6).

### 3.9 Direct healthcare cost

The total direct medical cost of RSV‐specific hospitalizations was €7.1 million, at a mean annual cost of €2.4 million. Most costs were generated by children aged <2 years (€6.8 million, 96.0%) and by previously healthy children (€6.2 million, 87.6%). Children aged <6 months accounted for €5.1 million (71.5% of the RSV‐specific hospitalization costs). RSV‐specific & Bronchiolitis cases generated costs of €11.1 million, of which 96.3% were related to children aged <2 years and 89.4% to previously healthy children. As for RSV‐specific & ALRI cases, they generated costs of €15.3 million, of which 87.7% were related to children aged <2 years and 84.8% to previously healthy children.

Mean (SD) and median (IQR) costs per patient are presented in Table 3. The mean (SD) cost per patient aged <5 years was €1004 (€1875) in RSV‐specific hospitalizations. Across all definitions, the highest mean costs per patient were observed in children with at least a risk factor and in children under 1 month of age.

## 4 DISCUSSION

Using an administrative data review from 2015 to 2018, we found a high burden in Portugal of hospitalizations potentially due to RSV in children aged <5 years, especially during the first year of life. Across all age groups, most cases were observed in previously healthy children.

We found hospitalization rates of 5.6, 9.4, and 11.8 per 1000 children aged <5 years and of 13.4, 22.5, or 25.9 in children aged <2 years, for RSV‐specific, RSV‐specific & Bronchiolitis, or RSV‐specific & ALRI codes, respectively. Results are consistent with findings from other studies using similar methodologies for Portugal and Spain,^14^, ^23^ for RSV‐specific and unspecified acute bronchiolitis case definitions.

Worldwide, substantial variation is found in hospitalization rates across studies, reflecting the use of distinct methodologies and clinical practices, in addition to potential geographic and seasonal variations.^1^, ^24^ Some studies consider acute bronchiolitis only, whereas others include acute respiratory infection (ARI) hospitalizations, often combined with laboratory data.^1^ A global annual RSV‐ARI hospitalization rate of 4.4 (95% CI 3.0, 6.4) per 1000 children has been estimated among children <5 years, 19.2 (95% CI 15.0, 24.5) among children <1 year and 20.0 (95% CI 9.7, 41.3) among children <6 months,^1^ which is closer to our lower bound estimate. However, most studies included only RSV‐confirmed cases, an approach which may also underestimate the true burden of RSV, unless systematic testing is performed upon admission.^25^ Rates obtained using active laboratory‐confirmed surveillance were found to be almost twice as high as those obtained using RSV‐specific ICD‐10 hospital discharge codes and more than three times the rate in children aged 2–4 years.^24^

In Portugal, a study of 6743 samples analyzed through the influenza surveillance system (2014–2018) in children aged <5 years found that 72.5% were positive for RSV.^20^ Bronchiolitis and pneumonia were significantly associated with RSV‐positive cases among children aged <5 years, which is consistent with our findings. Also, Hall et al. observed that bronchiolitis was the most frequent diagnosis in RSV‐positive inpatients aged <12 months (reported in 85% of cases), whereas in children aged between 2 and 5 years, pneumonia (51%) and acute asthma (60%) were the most frequent diagnoses.^9^ This supports the relevance of broader RSV case definitions, particularly for children aged >2 years.

Importantly, studies converge in the observation of the highest RSV hospitalization rates in the youngest age groups.^10^ We found that, across the different used definitions, children aged <2 years accounted for 88.4% to 96.4% of cases and children aged <6 months for 52.3% to 67.1%.

We found that most children hospitalized for RSV were healthy, which is consistent with previous studies, reporting >96% of cases in children without any risk factor.^14^, ^23^ In England, Murray et al. estimated that 85% of infants who are admitted to hospital with bronchiolitis are born at term, with no known predisposing risk factors for severe RSV infection.^8^ Differences may be explained by prematurity, which is underreported in our database—in our study, prematurity is registered in 1.2% of RSV‐specific cases, whereas between 2015 and 2021, the official country data indicates that the total percentage of preterm live births in Portugal ranged from 6.9% to 8.2%^26^—and was thus not included as a risk factor in the statistics. In the cohort analyzed by Murray et al., 7.5% of children were born preterm, rising to 14.6% in those admitted for bronchiolitis.^8^ The data on total children born preterm from Murray et al. are aligned with estimates published for England and similar to the rates reported for Portugal,^27^ and could thus potentially be used as a proxy for the percentage of children hospitalized for RSV who might have been born preterm.

LOS at the hospital is within reported ranges.^14^, ^23^, ^28^, ^29^ Stays were longer in younger children, with the highest LOS observed in children under 1 month of age, in accordance with previous findings.^30^ The study demonstrates a nonnegligible use of mechanical ventilation during RSV‐related hospitalizations (1.2% to 1.5% invasive and 6.4% to 8.2% noninvasive ventilation), potentially illustrating the cases requiring care at the ICU. In‐hospital mortality rate (0.1% to 0.2%) and characteristics of the deceased children are worrisome and in accordance with previous findings.^14^, ^23^

An annual cost of €2.4 million was estimated for RSV‐specific hospitalizations, mostly driven by previously healthy children and by those aged less than 2 years. These estimates may be just the tip of the iceberg as they include only direct costs and do not include patients treated in the private nor in the outpatient setting. Available international studies suggest that the incidence of RSV cases in the outpatient setting may be more than 30 times higher than the one observed in the inpatient setting.^9^ Additionally, indirect costs of lost productivity from parents and costs due to the morbidity caused by RSV on a long‐term basis were not considered. Still, results enable an understanding that in Portugal as in other countries, strategies aimed only at high‐risk children will have a limited ability in reducing the clinical and economic burden of RSV infection.

Furthermore, the RSV‐specific case definition is expected to underestimate the burden of RSV, due to the lack of routine RSV testing and coding practices. Using other case definitions, the estimated direct annual cost with hospitalizations would rise to €3.7 million for RSV‐specific & Bronchiolitis and up to €5.1 million for RSV‐specific & ALRI hospitalizations. The broad range of estimated costs with direct hospitalizations according to the used definition highlights the need for a universal RSV surveillance system to better understand the true burden of RSV in Portugal.

This is the first study reporting the burden of ALRI hospitalizations potentially related to RSV in Portugal including children up to 5 years old, considering distinct RSV definitions, and using data classified with ICD‐9 and 10. Access to full national NHS hospitalization data provides a comprehensive picture of the potential burden of RSV on families and the healthcare system. The estimated cost represents a very accurate vision of the hospitalization cost for the NHS, as it reflects the real funding value of the included NHS episodes.

The major limitation of the present study is that it relies on data from an administrative database which does not include a linkage to RSV laboratory testing. This might lead not only to the inclusion of RSV‐negative cases but also to the omission of RSV‐positive cases elsewhere classified. The available data do not enable us to quantify the proportion of the reported unspecified ALRI cases that have been caused by RSV and not by other agents, which may lead to an overestimation of RSV cases under the definition RSV‐specific & ALRI. RSV is widely reported as the most common pathogen identified in young children with ALRI (mainly pneumonia and bronchiolitis).^29^, ^31^, ^32^ However, other infectious agents can cause severe ALRI.^31^, ^33^ In children, bacterial pneumonia is usually caused by *Streptococcus pneumoniae* or by *Haemophilus influenzae type b* (Hib), where no systematic vaccination occurs and RSV is the most common viral cause of pneumonia.^31^, ^33^ Shi et al. performed a systematic review and meta‐analysis, which supports that RSV, influenza (IFV), parainfluenza (PIV), human metapneumovirus (HMPV), and rhinovirus (RV) are important causes of ALRI in young children, and have estimated the percentage of severe ALRI attributable to each virus to be 90%, 80%, 70%, 73%, and 30%, respectively.^34^ Although these viruses have specific ICD codes, they are also likely to be under‐reported and could be included under our unspecified ALRI cases.

Data are also subject to coding errors or missing information, such as full data on newborns or on underlying medical conditions that may not be coded in the analyzed episodes. Further research is needed to clarify the specificity and sensitivity of ICD‐9 and 10 codes in the Portuguese NHS setting and to assess the real presence of risk factors, namely, those related to prematurity, birth weight, and exposure to tobacco, which are expected to be underestimated in administrative data. Regarding cost per patient, data are presented both as arithmetic mean (SD) and as median (IQR) in the tables, but we focus on the mean cost when reporting the results, since, despite the nonnormal distribution of these variables, arithmetic mean provides the information required for healthcare policy decisions, as it informs about the total cost that will be incurred by treating all patients.^35^

## 5 CONCLUSION

RSV is a relevant driver of hospitalizations among children in Portugal. Children are at greater risk of hospitalization during the first year of life, although a nonnegligible number of hospitalizations was also observed in older children. Most cases are observed in previously healthy children. Being born right before or during the epidemic season seems to increase the risk of hospitalization, but children born during the summer months also are at risk. These findings highlight the need for an effective RSV surveillance system, well‐defined prevention strategies, and a new preventative solution that could help extend protection to all infants.

## CONFLICTS OF INTEREST

The study was funded by Sanofi and conducted by IQVIA. Carmo M and Lopes H are IQVIA employees. Gomes C, Martins M, Guzman C, and Bangert M are Sanofi employees and may hold shares and/or stock options in the company. Bandeira T, Rodrigues F, Januário G, Tomé T, and Azevedo I have received fees from Sanofi associated with the study elaboration, but the authors were not paid for writing the publication.

## AUTHOR CONTRIBUTIONS

**Teresa Bandeira:** Conceptualization; investigation; methodology; supervision; validation; writing‐review and editing. **Mafalda Carmo:** Conceptualization; data curation; formal analysis; investigation; methodology; project administration; resources; visualization; writing‐original draft; writing‐review and editing. **Hugo Lopes:** Conceptualization; data curation; formal analysis; investigation; methodology; project administration; resources; software; writing‐review and editing. **Catarina Gomes:** Conceptualization; funding acquisition; investigation; methodology; project administration; resources; supervision; validation; visualization; writing‐review and editing. **Margarida Martins:** Conceptualization; funding acquisition; investigation; methodology; project administration; validation; writing‐review and editing. **Carlos Guzman:** Conceptualization; funding acquisition; investigation; methodology; project administration; writing‐review and editing. **Mathieu Bangert:** Conceptualization; funding acquisition; investigation; methodology; project administration; resources; supervision; validation; visualization; writing‐review and editing. **Fernanda Rodrigues:** Conceptualization; investigation; methodology; writing‐review and editing. **Gustavo Januário:** Conceptualization; investigation; methodology; writing‐review and editing. **Teresa Tomé:** Conceptualization; investigation; methodology; writing‐review and editing. **Inês Azevedo:** Conceptualization; investigation; methodology; writing‐review and editing.

## ETHICAL APPROVAL AND PATIENT CONSENT STATEMENT

Data were provided anonymized from ACSS and may be used for research purposes without ethics committee approval or informed consent. In addition, the protocol of the Burden of Acute Respiratory Infections (BARI) study was validated by a panel of clinical experts, classified by the Agency of Medicines and Medical Devices (AEMPS) as an observational study, and approved by the Ethics Committee of Hospital Clinic de Barcelona (HCB/2020/1132), who waived the need for participant consent.

## PERMISSION TO REPRODUCE MATERIAL FROM OTHER SOURCES

Not applicable.

### PEER REVIEW

The peer review history for this article is available at https://publons.com/publon/10.1111/irv.13066.

## Supporting information


**Table S1.** List of analysed risk factors and ICD9/10 codes used
**Table S2.** List of ICD‐9 and ICD‐10 codes used as “severity marker”

**Figure S1.** Share of cases where patients had a risk factor, per risk factor

**Figure S2.** Share of cases with a respiratory “severity marker”

## Data Availability

The data that support the findings of this study are available from IQVIA, but restrictions apply to the availability of these data, which were used under license for the current study, and so are not publicly available. Data are however available from the authors upon reasonable request and with permission of IQVIA.
