# Supplementary figures and images for: Burden and severity of children's hospitalizations by respiratory syncytial virus in Portugal, 2015–2018
Source: Influenza Other Respir Viruses. 2022 Nov 14;17(1):e13066. doi: 10.1111/irv.13066 (PMC9835409; doi:10.1111/irv.13066)

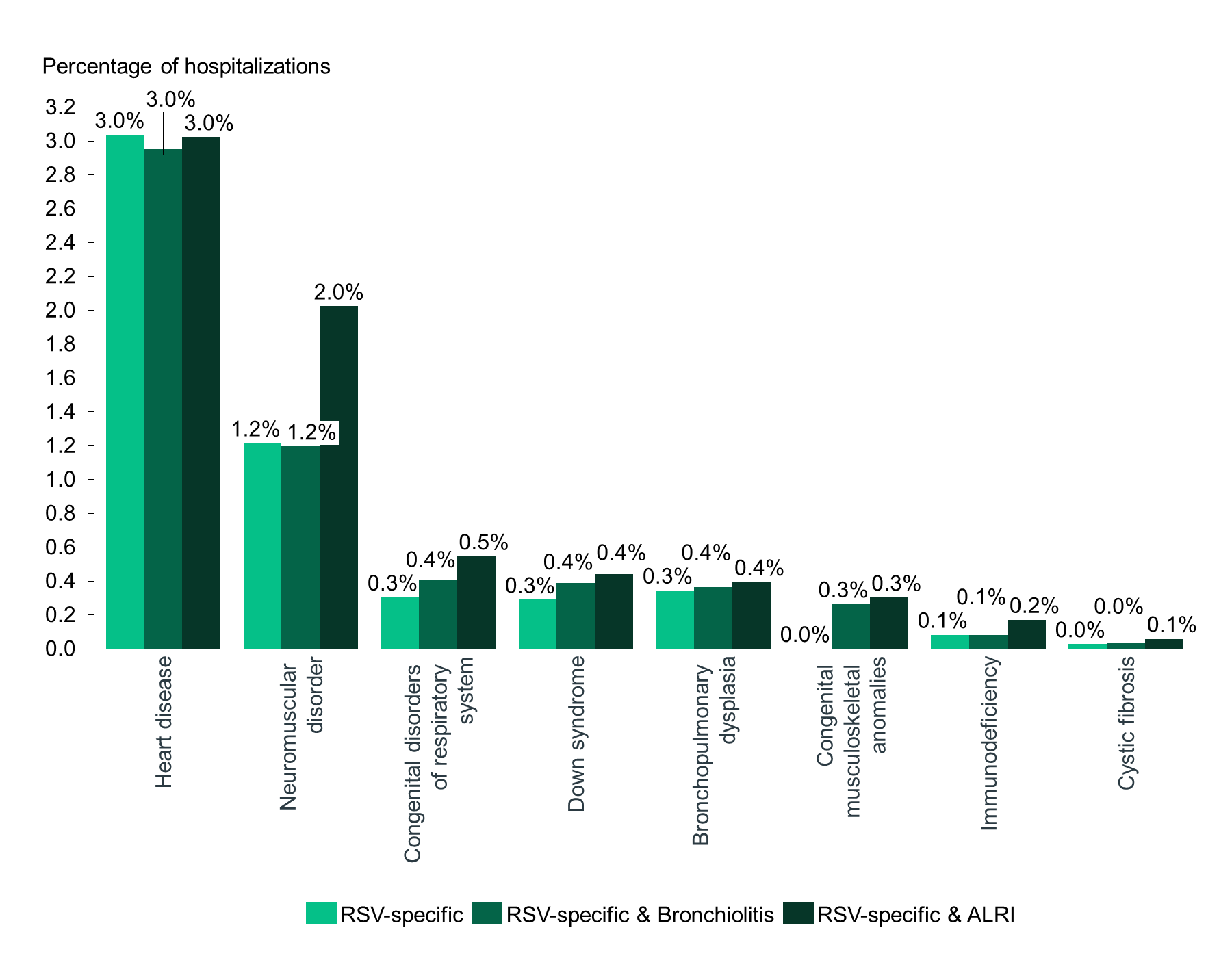

Supplement: Supplementary file 2 — Figure S1. Share of cases where patients had a risk factor, per risk factor [file IRV-17-e13066-s002.tif]

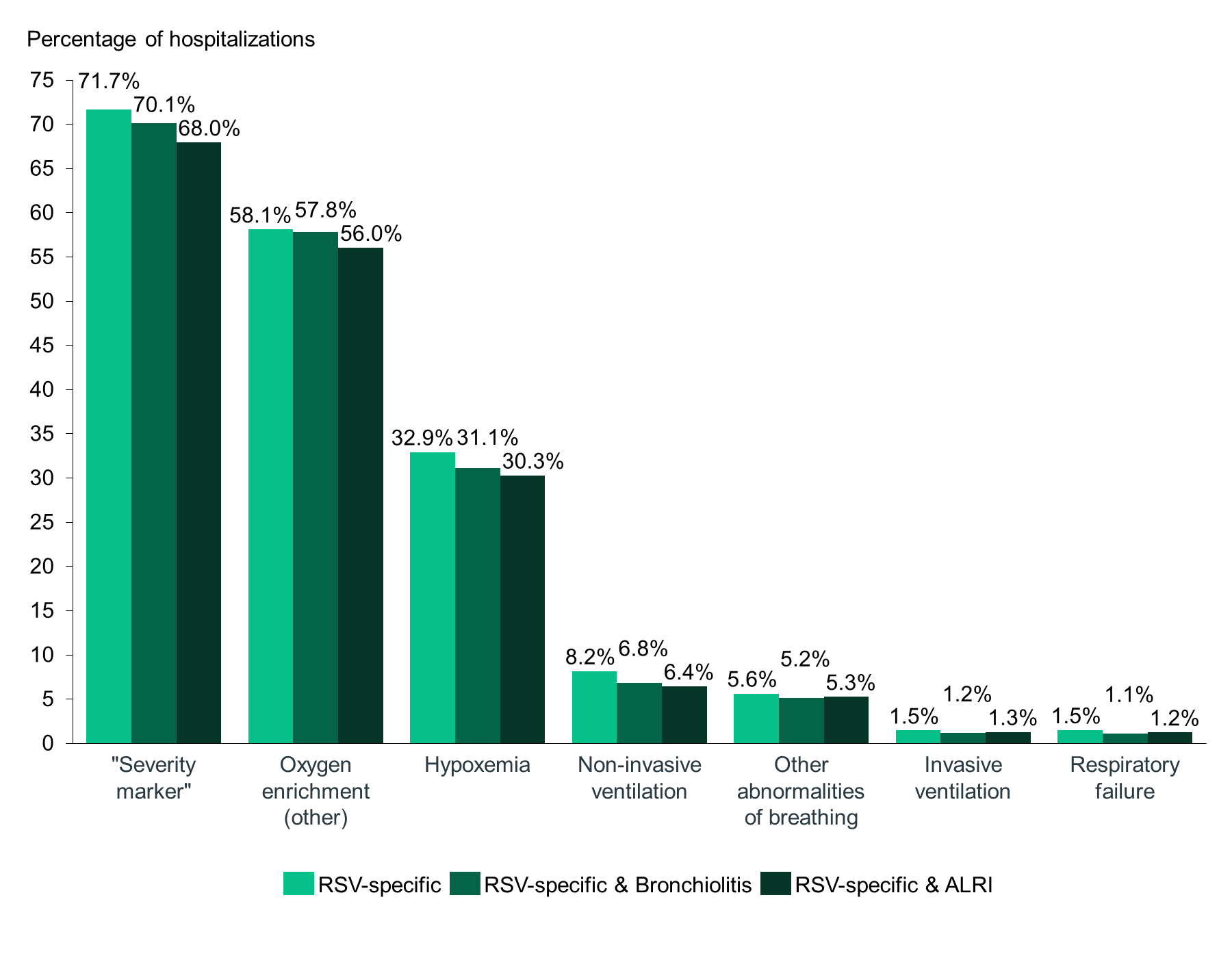

Supplement: Supplementary file 3 — Figure S2. Share of cases with a respiratory “severity marker” [file IRV-17-e13066-s003.tif]
